# Zinc oxide nanoparticles supplementation improve the quality and lipid profiles of frozen boar semen

**DOI:** 10.3389/fvets.2026.1836234

**Published:** 2026-05-18

**Authors:** Kangmin Li, Yongqi Chen, Yongsheng Fang, Shanben You, Xinyue Wan, Zixuan Zhuang, Yunhai Zhang, Zubing Cao

**Affiliations:** Anhui Province Key Laboratory of Local Livestock and Poultry, Genetical Resource Conservation and Germplasm Innovation, College of Animal Science and Technology, Anhui Agricultural University, Hefei, China

**Keywords:** frozen semen, lipidomics, pigs, sperm quality, ZnONPs

## Abstract

Oxidative stress frequently compromises the quality of frozen-thawed sperm. Zinc oxide nanoparticles (ZnONPs) have demonstrated antioxidant effects on frozen semen from species such as goats, camels, and buffaloes; however, their impact on boar semen remains unclear. This study investigated the effects of ZnONPs supplementation in cryopreservation extenders (0, 0.05, and 0.1 mg/ml) on the frozen-thawed semen of Huoshou black boars. Compared with the control, the 0.1 mg/ml ZnONPs group significantly improved sperm motility and kinematic parameters after 0 and 1 h of incubation at 37 °C (*P* < 0.05). Furthermore, this group exhibited significantly greater sperm viability, plasma membrane integrity, and total antioxidant capacity (T-AOC), along with lower reactive oxygen species (ROS) levels (*P* < 0.05). Lipidomic analysis revealed 74 differentially expressed lipids in positive ion mode (8 upregulated and 66 downregulated) in the ZnONPs-supplemented group. These findings indicate that supplementation with 0.1 mg/ml ZnONPs improves the cryopreservation outcomes of boar semen.

## 1 Introduction

Cryopreservation technology facilitates genetic improvement and ensures a continuous supply of high-quality semen for artificial insemination (1). This approach enhances the utilization efficiency of superior genetics, enables cross-regional exchange (2), and addresses challenges such as the geographical limitations of ambient semen transport and suboptimal fertilization due to asynchronous gamete maturation (3). However, boar sperm are particularly susceptible to cryodamage (4). During cryopreservation, sperm are significantly affected by oxidative stress (5), leading to a marked decline in semen quality (6). Studies indicate that sperm generate substantial amounts of ROS (7, 8). When excessive ROS overwhelm the limited antioxidant defenses of sperm, they cause membrane damage, lipid peroxidation, and DNA fragmentation (9). Temperature fluctuations during freezing can alter sperm lipid composition and impair membrane function. The use of cryoprotectants in semen extenders is essential to mitigate freezing damage; omitting them increases the risk of sperm injury (10).

Zinc plays a critical role in sperm function by regulating antioxidant defense and oxidative stress, thereby preserving membrane integrity and motility (11). As a bioavailable source of zinc, zinc oxide exerts similar effects, although excessive exposure may adversely affect reproductive function and sperm quality. ZnONPs are inorganic nanomaterials with particle sizes less than 100 nm. They possess strong antioxidant (12, 13), free radical scavenging (14), and antistress properties (15), as well as high safety (16). Consequently, ZnONPs have attracted considerable research interest. For example, Fayez Eman et al. reported that 0.1 mg/ml ZnONPs improved the physiological function and antioxidant capacity of cryopreserved dog semen (17). Mohammad Javad Karimi Sabet et al. reported that an appropriate concentration of ZnONPs enhanced the quality of frozen chicken semen (18). Similarly, Sultan T et al. demonstrated that ZnONPs reduce oxidative stress and improve postthaw sperm quality in horses (19). While ZnONPs have beneficial effects on the cryopreservation of semen from dogs, chickens, and horses, their impact on boar semen remains unexplored.

This study examined the antioxidant effects of ZnONPs on the cryopreservation of Huoshou black boar semen. These findings provide a theoretical basis and technical support for improving cryopreservation protocols, optimizing extender formulations, and promoting the application of cryopreservation in pig reproduction.

## 2 Materials and methods

Except as otherwise indicated, the experimental reagents employed in this study were obtained from Sigma–Aldrich (St. Louis, Missouri, USA). As otherwise specified, the ZnONPs mixture was utilized at a concentration of 0.1 mg/ml.

### 2.1 Preparation of chemicals

For the current investigation, ZnONPs were acquired via commercial channels from Sigma-Aldrich (Product #544906, PubChem CID: 24878793). ZnONPs were characterized as follows (20): their average diameter was 30.92 ± 1.25 nm and a positive zeta potential of 32.16 ± 0.252 mV. These values were in accordance with the manufacturer's specifications. A working suspension was prepared by dispersing ZnONPs with purified water, yielding a stock solution at 1 mmol/L. A 1-gram aliquot of cryopreserved semen extender (Green Auris, Beijing, China)—which contains core ingredients such as glucose, sucrose, sodium bicarbonate, citric acid, sodium citrate, antioxidant compounds, surface-active agents, and cryoprotective additives—was homogenously combined with 20 ml of ultrapure water, followed by preheating to 37 °C in preparation for subsequent experimental procedures. It is important to note that the exact formulation of this commercially available product is proprietary, and thus, its full composition has not been made public by the supplier. For the control cohort, the thawing medium consisted of the base formulation without ZnONPs. All the samples from both the control and ZnONPs-supplemented groups were subjected to a minimum of three replications.

### 2.2 Animals, semen collection, and sample processing

Four adult Huoshou black boars were housed under standardized husbandry practices: regulated environmental temperature, consistent dietary protocols, a 16-h light cycle that combines natural and artificial illumination, and unlimited access to potable water. The fraction of ejaculate with high gamete density was collected from the boars using a gloved-hand technique. The fraction of ejaculate with high gamete density was collected from the boars using a gloved-hand technique: a trained technician donned sterile latex gloves, initiated manual stimulation of the animal's penile tissue, carefully grasped the spiral glans upon the boar's erection, and after discarding the initial 10–15 ml (which contained a low density of the target gametes), this high-density fraction was transferred into a sterile vessel preheated to 37 °C. Each boar had semen collected twice weekly, with three of their ejaculates set aside for freezing sperm. Ejaculate samples were obtained from each individual boar on two separate occasions per week. From every collection, three ejaculates were allocated for sperm cryopreservation. Each animal underwent a total of eight collections over the course of the study. For the purposes of this research, “semen” stands for the entire ejaculate, which includes spermatozoa and seminal plasma, whereas “sperm” indicates only spermatozoa that have been separated from seminal plasma. For the freezing process, only top-quality semen, defined by a milky white color, freedom from any foreign particles or unusual smells, and a motility rate of over 80%, was chosen.

### 2.3 Cryopreservation of semen

The freezing process involved two stages (21): semen from different Huoshou black boars was first pooled for the purpose of reducing interanimal variability. Then, an appropriate amount of ZnONPs (pig frozen semen dilution kit purchased from Beijing Yuantian Aori Company) was added to semen dilution solutions I and II. Qualified semen samples (whole ejaculates) were first proportionally diluted with predilution solutions and transferred to a 17 °C controlled environment for preliminary cooling, which lasted approximately 4 h. After the sample achieved equilibrium at 17 °C, the ejaculate was subjected to centrifugation using a large-capacity centrifuge at 800 × g for a 15-min period. The seminal plasma fraction (the resulting supernatant) was removed and discarded, and the concentrated pellet of the reproductive cells was quickly resuspended in a commercially sourced cryopreservation medium (provided by Green Auris, Beijing, China). This resuspended ejaculate preparation was placed in a pre-adjusted 17 °C water bath prior to being transferred to a temperature-controlled environment held at 4 °C for a secondary cooling step; this phase persisted for approximately 4 h. Once equilibrium was achieved at 4 °C, isothermal cryoprotection solution (Green Auris, Beijing, China) consisting of solution I (265–280 ml), glycerol (15–30 ml), and surfactant (mass fraction 0.1–0.4%) was added to the semen. The main components of Solution I are glucose (22–30 g), citric acid (2.5–4.5 g), sodium citrate (4–8 g), HEPES sodium (2–3.2 g), Tris (4–7 g). EDTA-Na2 (1.5–4 g), sodium bicarbonate (0.5–2 g), cysteine (0.01–0.2 g), pure water (1,000 ml), and yolk fluid (250 ml) were added to 0.5 mL cryogenic straws (Minitube, Munich, Germany), which were sealed with sealing powder. After the semen was filled into the straws, the thin tubes were placed on the tube rack, and after placing them, they were placed in a foam container filled with liquid nitrogen for fumigation. The distance between the thin tubes and the surface of the liquid nitrogen was 3 cm. The semen was frozen and stored in liquid nitrogen for at least 2 months before being used in the study.

### 2.4 Design of the experiment

This investigation consisted of three experimental cohorts, with each cohort administered ZnONPs at concentrations of 0 mg/ml, 0.05 mg/ml, or 0.1 mg/ml; each cohort also included three independent experimental replicates. Each pre-formulated thawing solution was heated in advance using a 37 °C water bath over a 10-min duration prior to its application for thawing cryopreserved ejaculate samples. Once the straw that had been frozen was removed from liquid nitrogen, it was promptly put into a 50 °C water bath and shaken vigorously for 15 s. The outer surface of each straw was then dried with sterile absorbent paper. Next, the sealed part at one end of every straw was opened by cutting, after which the internal semen was squeezed into the thawing solution that had been prepared (22). This semen underwent thorough mixing and was then set aside for use in subsequent tests.

### 2.5 Evaluation of sperm motility and kinematic parameters

For determination of the optimal ZnONPs dose, sperm motility along with related dynamic parameters post cryopreservation-thawing were evaluated following 37 °C incubation at 0, 1, 2, and 4 h. Motility and associated kinetic parameters of sperm were measured using a computer-assisted sperm analysis (CASA) system (ZhuXianzi, Guangzhou, China), and this equipment was fitted with a viewing apparatus featuring trinocular phase-contrast functionality, a temperature-regulated zone, and a digital imaging component. For each sample, eight sperm dynamic indices were evaluated by analyzing five fields chosen at random (with no fewer than 200 spermatozoa present in every field): motility, average path velocity (VAP, μm/s), straightness (STR, %), wobble (WOB, %), amplitude of lateral head (ALH, μm), curvilinear velocity (VCL, μm/s), straight-line velocity (VSL, μm/s), and linearity (LIN). Default specifications encompassed 60 frames per second; minimum captured frames: 21; and spermatozoa with a VAP ≥ 10 μm/s were classified as motile.

### 2.6 Assessment of sperm viability

Sperm viability was detected via trypan blue staining (23). A 0.2 g aliquot of trypan blue was precisely measured using an electronic balance (manufactured by Shanghai Hooran Electronics, China) and transferred into a 15 ml centrifuge tube (supplied by Corning, USA). Deionized water was added to bring the mixture to a total volume of 5 ml, and the tube was shaken repeatedly until the dye dissolved fully. To obtain a 0.4% trypan blue solution, a 10 μl pipettor was used to obtain 5 μl of the 4% trypan blue storage solution. Then, 45 μl of pure water was added, and the mixture was mixed well. The prepared trypan blue working solution, slide, and cover slip were preheated in a 37 °C environment for 10 min. Fifty microliters of the control sample and 50 microliters of the frozen semen (with 0.10 milligrams per milliliter of ZnONPs added) were mixed with the prewarmed trypan blue working solution in an equal volume ratio (1:1). Then, the resulting mixture was incubated at 37 degrees Celsius for 15 min. A 10-μl portion of the incubated mixture was dispensed onto a glass slide, and a cover slip was applied; the preparation was then examined and quantified using a 400 × light microscope (produced by Nikon, Japan). A sperm with a transparent head was defined as live sperm, and a sperm with an opaque head was defined as dead sperm. The percentage of live sperm reflects sperm viability, with relevant data recorded, and the experiment was repeated three times.

### 2.7 Evaluation of sperm morphology

Eosin staining (E8090, Solarbio, Beijing, China) was employed to detect morphological abnormalities in the sperm. There are three main categories of sperm abnormalities. The first type includes abnormalities in sperm head morphology, such as overly large heads, overly small heads, conical heads, pear-like heads, and duplicated heads, among other variations. The second type involves issues with the neck and midsection, including curved necks, thickened and poorly formed midsections, and excessively slender midsections. The third type includes abnormalities of the sperm tail, such as abnormally short tails, extra tails, and tail breakages. The cell density was adjusted to 2 × 10^4^ cells per ml. Subsequently, 10 μl of each seminal sample was deposited onto a glass slide, and a thin cellular film was created by placing a coverslip over the sample before it was left to air dry naturally at ambient temperature. After the thin film had dried completely, the slide was immersed in methanol for fixation. 10 min later, the slide was removed, and the sample was allowed to air dry again. Next, the samples were stained with a 2% eosin solution at room temperature for 15 min, followed by gentle rinsing of both slide surfaces with deionized water. Cellular morphology and irregularity frequency were then evaluated and enumerated via a light microscope at 400 × magnification.

### 2.8 Evaluation of plasma membrane integrity

The integrity of the sperm plasma membrane was detected via the hypoosmotic swelling test (24). The concentration of the sperm cells was set to 1 × 10^6^ per ml. To construct the hypotonic solution, 0.735 g of sodium citrate (Source Leaf Biology, S11111) and 1.351 g of fructose (Sigma, F3501) were weighed and dissolved in 100 mL of pure water. After thorough dissolution, the solution was filtered through a 0.22 μm filter. Next, 1 mL of thawed semen was incubated with 9 mL of hypotonic solution at 37 °C for 30 min. Throughout this incubation, the centrifuge tube was gently agitated every 5 minutes. Once the incubation was complete, 10 μl of the processed semen was collected and placed on a glass slide, and the sample was covered with a coverslip. The morphology of the sperm tails was then assessed via a 400 × light microscope.

### 2.9 Analysis of DNA integrity

Acridine orange (AO) staining (S19094; Shanghai Yuanye Bio-Technology Co., Ltd., Shanghai, China) was utilized to assess DNA structural intactness (25). The cell density was standardized to 2 × 10^4^ cells per milliliter. Subsequently, 10 μL aliquots of each seminal specimen were uniformly spread on glass slides and allowed to air-dry naturally. Specimen slides were fixed in a 3:1 (v/v) absolute ethanol-glacial acetic acid mixture for 5 min prior to 10 min of staining with freshly prepared AO solution. After staining, extensive rinsing with distilled water preceded air-drying at ambient temperature. Specimens were examined using a fluorescence microscope (Olympus, Tokyo, Japan) operated at 400 × magnification; excitation and emission wavelengths were configured to 480 nm and 530 nm, respectively. A total of 200 cells were selected and analyzed from no fewer than three separate microscopic fields. Cells possessing undamaged DNA displayed green fluorescence in their head region. Counting these fluorescent cells allowed for the quantification of DNA structural intactness.

### 2.10 Analysis of acrosome integrity

These experiments followed the procedures outlined earlier (26). Specifically, 20 μl of PNA-FITC (Sigma, L7381) was added to 180 μl of 1 × PBS. The control group and frozen seminal samples treated with 0.10 mg/ml ZnONPs underwent washing procedures, after which the cell density was standardized to 1 × 10^6^ cells/ml. 20 microliters of each processed sample was then deposited onto glass slides, followed by air drying at ambient temperature. The slides were subsequently submerged in anhydrous formaldehyde for 10 min and allowed to air dry once more. 200 microliters of PNA-FITC solution was added to dilute the residual droplets on the slide surfaces, and this dilution process was carried out at 37 °C in a 5% CO_2_ atmosphere under dark conditions for a 30-min incubation period. After incubation, the slides were rinsed softly 2–3 times with PBS, allowed to air dry, and then covered with a coverslip for mounting. Imaging was conducted promptly via a fluorescence microscope (Olympus, Japan) at 400 × magnification, with the entire experimental process executed under dark conditions to avoid fluorescence quenching. The experimental data were recorded systematically, and the full protocol was conducted in triplicate to ensure reproducibility.

### 2.11 Assay of mitochondrial activity

Rh123-PI staining was employed to assess mitochondrial functional activity (27). An aliquot of Rh123 (0.0004 g) was weighed, dissolved in 2 mL of DMSO, passed through a 0.45 μm filtration membrane (Millipore, USA), and preserved at −20 °C under light-protected conditions. The PI stock solution was removed from refrigerated storage, brought to a 2 mg/ml concentration using PBS, and then stored in light-protected environments for subsequent experimental use. The target cell density was normalized to a range of 3–6 × 10^6^ cells per milliliter. 100 individual 1.5 ml centrifuge tubes filled with PBS (each corresponding to one of the 100 test specimens) were heated in advance in a 37 °C constant-temperature water bath (manufactured by Shanghai Constant Scientific Instruments, China) over a 10-min duration. One microliter of PI and 1 μl of Rh123 were incorporated into the prewarmed PBS, and the mixture was subsequently incubated at 37 °C under light-protected conditions for a 10-min duration. After this incubation step, 50 μl of each seminal sample was added, and the mixture was incubated at 37 °C in a dark, humid environment for 30 min. 10 microliters of each treated sample was placed on a glass slide and covered with a coverslip. Specimens were then visualized with a microscope at 400 × magnification, and 200 cells were enumerated per sample. Fluorescence microscopy revealed two cell populations: those with intact mitochondrial membrane function presented red fluorescence in the head's nuclear region alongside green fluorescence in the caudal segment. Cells displaying red fluorescence in the head's nuclear area without corresponding caudal fluorescence were categorized as nonviable. The percentage of cells exhibiting green fluorescence reflects the degree of mitochondrial membrane integrity. The data were documented, and the entire protocol was conducted in triplicate.

### 2.12 ROS content assay

The ROS levels were quantified via an ROS detection kit (catalog no. S0033S; Shanghai Biyuntian Biotechnology Co., Ltd.). Initially, the cell density across the two experimental groups was standardized to a final concentration of 1 × 10^6^ cells per milliliter. All ensuing experimental procedures were executed in full accordance with the operational guidelines supplied by the kit manufacturer. Specifically, 10 μl of DCFH-DA stain solution was introduced to each cell specimen to enable labeling of intracellular ROS. Once staining was finalized, measurements were conducted using a fluorescence microplate reader: the excitation wavelength was set to 488 nm, the emission wavelength was set to 525 nm, and the optical density (OD) reading for each sample well was recorded under these settings.

### 2.13 Detection of the MDA content

A malondialdehyde kit (A003-1, TBA method), which was produced by Nanjing Jiancheng Bioengineering Institute, was used for detection (28). After the fresh semen was diluted by a factor of 10, 2 millilitres of each extract were separately dispensed into centrifuge tubes. After the semen was removed via centrifugation, 2 millilitres of frozen PBS was added, and ultrasonic treatment was used to lyse the cells to release MDA. The cells were then spun at 12,000 rpm for 5 min at 4 °C, after which the supernatant was collected for subsequent use. Four 15-milliliter centrifuge tubes were collected, which were labeled the blank tube, standard tube, test tube, and control tube. Reagents were added according to the instructions. Small holes were made on the covers of the four tubes, which were then placed in a 98 °C water bath. The OD value of each well was subsequently read via an enzyme detector at 532 nm. The MDA content in the samples was calculated according to the formula.

### 2.14 T-AOC activity assay

T-AOC determination was conducted using a commercial detection kit (Catalog No. A015-2; ABTS rapid method, 100 tests) from Nanjing Jiancheng Bioengineering Institute (29). Prior to analysis, frozen seminal specimens were thawed, followed by 10-fold dilution in an appropriate buffer; 2 ml portions of each diluted sample were then aliquoted into centrifuge tubes. After centrifugation for cell pelleting and supernatant discarding, 2 ml ice-cold PBS was introduced for resuspension of the cell pellet. The cell suspension was sonicated in an ice bath to lyse the cell membranes and liberate the intracellular antioxidant components. Following sonication, the sample mixture underwent centrifugation at 12,000 rpm rotational speed, with temperature regulated at 4 °C for a 5-min interval. The resulting supernatant, containing the extracted antioxidants, was collected and preserved for subsequent T-AOC determination. For the microplate-based assay, blank control wells were loaded with 10 μl of distilled water, 20 μl of Reagent IV working solution, and 170 μl of ABTS working solution. In contrast, each standard well received 10 μl of Trolox standard solution (at varying concentrations), 20 μl of Reagent IV working solution, and 170 μl of ABTS working solution. All wells underwent incubation at ambient temperature for a 6-min period to promote reaction progression, followed by measurement of per-well OD values at 405 nm using a microplate reader. Finally, the T-AOC value of each seminal sample was derived by referencing the standard curve constructed from the Trolox standard solutions.

### 2.15 Detection and analysis of lipid molecules in frozen semen

A 90% Percoll solution was prepared as a 9:1 volumetric blend of undiluted Percoll and 10 × PBS. Next, an equal volume of this 90% Percoll mixture was blended with 1 × PBS to create a 45% Percoll solution, and both solutions were kept at 4 °C for later use. Three tubes of frozen semen were selected from each of the experimental groups and the control group. These cryopreserved semen specimens were thawed in 15 ml centrifuge tubes with thawing medium, followed by centrifugation at 3,000 rpm over a 10-min interval. Following centrifugation, the supernatant was discarded; 2 ml of 1 × PBS was then added to the resulting pellet for cell resuspension. For density gradient centrifugation preparation, 10 ml of 90% Percoll was initially placed at the base of a 50 ml centrifuge tube. Subsequently, 10 ml of 45% Percoll was gently layered over the 90% Percoll fraction at a slow pace. Once the density gradient stabilized, the pretreated sperm sample was gently loaded onto the top of the 45% Percoll layer to maintain the integrity of the separation column and prevent disruption of the liquid interfaces. The centrifuge tube contained the density gradient, and the sperm sample was placed into a centrifuge and then spun at 300 × g at 17 °C for 25 min. Following careful retrieval from the centrifuge, the enriched lower cell layer was pipetted and transferred into a 2 ml centrifuge tube. An equivalent volume of 1 × PBS was added to the enriched cell suspension, which was thoroughly mixed prior to centrifugation at 500 × g for 3 min for washing. The washed cell pellet was subsequently transferred into a cryovial. For chromatographic determination of the target analytes, a Vanquish ultrahigh-performance liquid chromatograph (Thermo Fisher Scientific) coupled to a Phenomenex Kinetex C18 column (2.1 × 100 mm, 1.7 μm) was utilized. Mobile phases included phase A (40:60 v/v water/acetonitrile supplemented with 10 mmol/L ammonium formate) and phase B (10:90 v/v acetonitrile/isopropanol, with 50 ml 10 mmol/L ammonium formate aqueous solution added to each 1,000 ml of the mixed solvent). Gradient elution was conducted via the following program: phase B held at 40% for 0–1 min, ramped linearly to 100% from 40% over 1–12 min, kept constant at 100% for 12–13.5 min, decreased linearly from 100 to 40% over 13.5–13.7 min, and ultimately held at 40% for 13.7–18.0 min. During the entire analytical procedure, all the samples were stored in an autosampler set to 10 °C. To reduce interference from variations in the instrument's detection signal, the samples were analyzed consecutively in a random sequence.

### 2.16 Statistical analysis

Sperm motility and related motility parameters were analyzed via one-way analysis of variance (ANOVA) followed by Tukey's *post hoc* test when appropriate. All other experiments were statistically analyzed via Student's *t*-test. Analyses were performed via GraphPad Prism 5.0 (GraphPad Software, San Diego, CA, USA). Data are expressed as the mean ± standard error of the mean (S.E.M.). A *p*-value less than 0.05 was considered statistically significant.

## 3 Results

### 3.1 Influence of ZnONPs on post-thaw sperm motility and kinematic parameters

**Figure 1:**
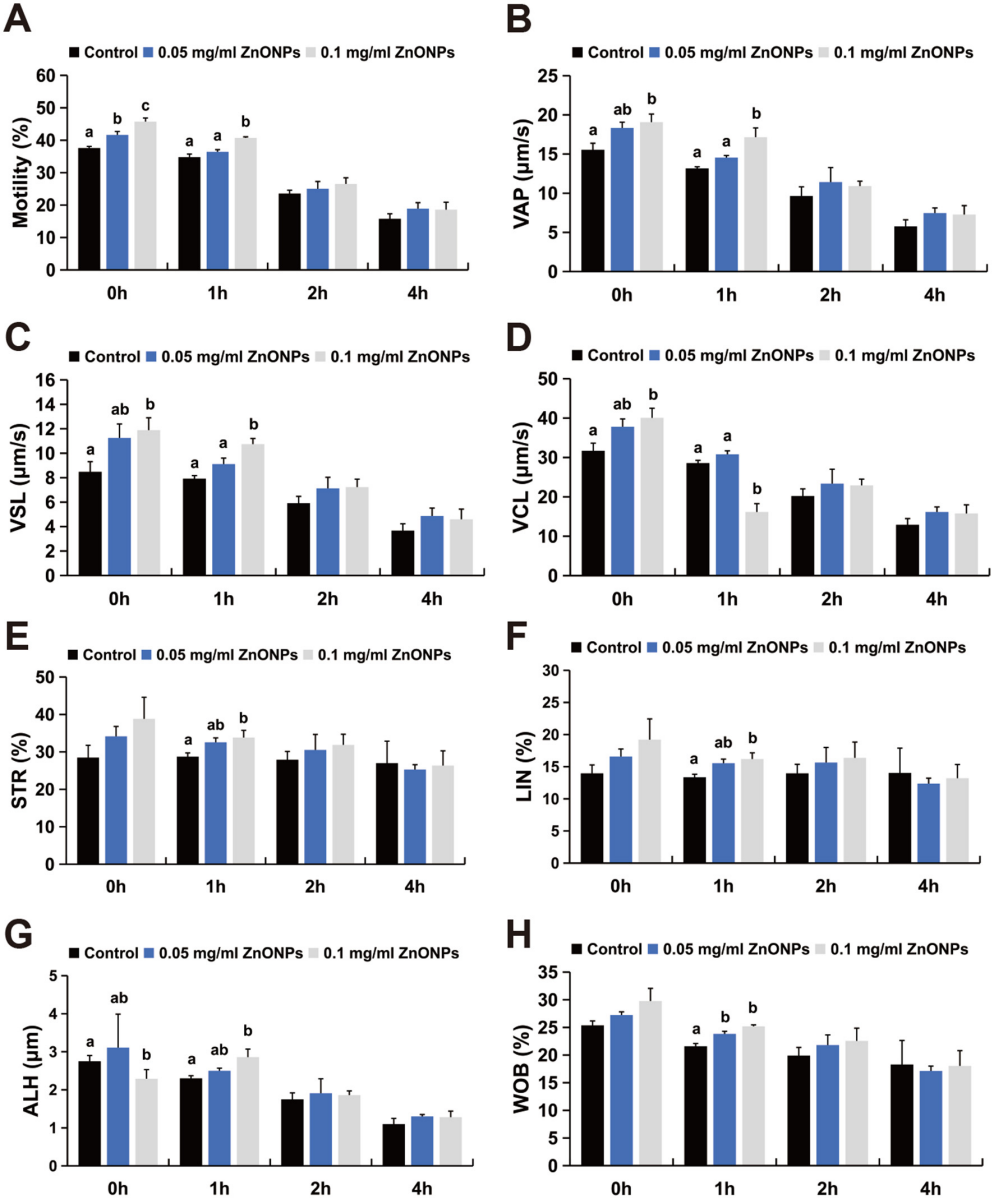
Influence of ZnONPs on Post-Thaw Sperm Motility and Kinematic Parameters. **(A)** Motility. **(B)** Average path velocity (VAP). **(C)** Straight-line velocity (VSL). **(D)** Curvilinear velocity (VCL). **(E)** Straight-Line Velocity. **(F)** Linearity (LIN). **(G)** Amplitude of lateral head displacement (ALH). **(H)** Wobble (WOB). The number of biological replicates was 4 boars, with 8 independent ejaculates collected from each. Data from at least three independent experiments were analyzed via one-way ANOVA and Tukey's *post hoc* test. Bars with different superscript lowercase letters (a, b, c) represent significant differences between groups (*P* < 0.05), whereas bars sharing a common letter or with overlapping letters (ab) represent no significant differences (*P* > 0.05).

The sperm were evaluated following incubation at 37 °C for 0–4 h post-thaw (Figure 1). Immediately after thawing (0 h), sperm motility was significantly greater in the group treated with 0.05 mg/ml ZnONPs than in the control group (Figure 1A, *P* < 0.05), although no significant differences in other kinematic parameters were noted. Compared with the control, treatment with 0.1 mg/ml ZnONPs led to significant increases in motility, VAP, VSL, VCL, and ALH (Figures 1A–D, G, *P* < 0.05). After 1 h of incubation, the WOB increased in the 0.05 mg/ml group (Figure 1H, *P* < 0.05). In contrast, the 0.1 mg/ml group demonstrated significant improvements in multiple parameters, including motility, VAP, VSL, VCL, STR, LIN, ALH, and WOB (Figures 1A-H, *P* < 0.05). No statistically significant differences were detected at the 2 or 4 h time points.

### 3.2 Impact of ZnONPs on the survival and morphological abnormality rates of frozen-thawed sperm

**Figure 2:**
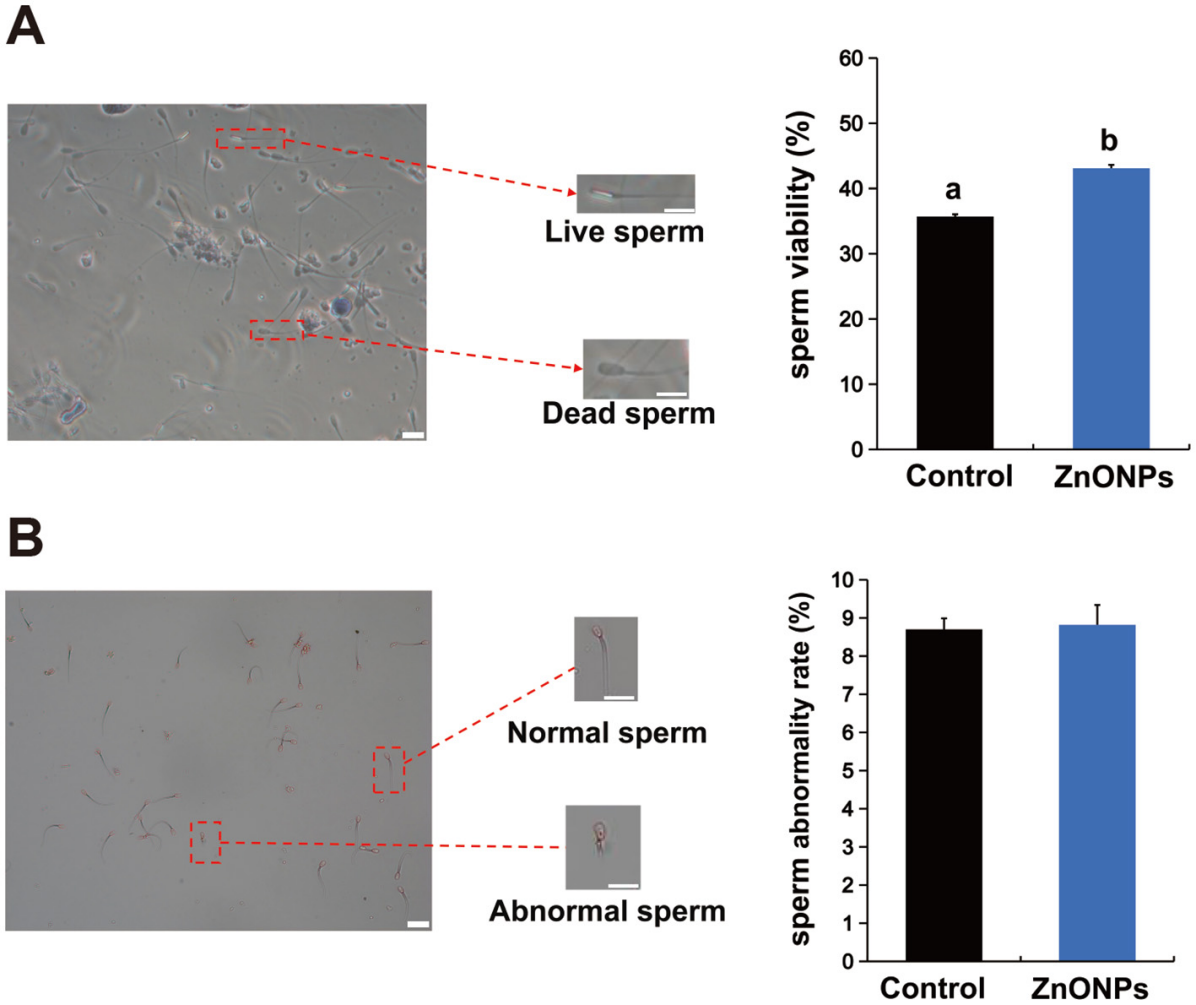
Impact of ZnONPs on the Survival and Morphological Abnormality Rates of Frozen-Thawed Sperm **(A)** Survival rate comparison between the control and experimental groups. Scale bar: 20 μm. **(B)** Abnormality rate comparison. Scale bar: 20 μm. The number of biological replicates was 4 boars, with 8 independent ejaculates collected from each. Data were analyzed via Student's *t*-test. Bars with different superscript lowercase letters (a, b) represent significant differences between groups (*P* < 0.05).

The sperm survival rate was significantly higher in the 0.1 mg/mL ZnONPs-supplemented group than in the control cohort (Figure 2A, *P* < 0.05). Conversely, the sperm morphological abnormality rate exhibited no significant variation across the two groups (Figure 2B, *P* > 0.05).

### 3.3 Effects of ZnONPs on the functional attributes of cryopreserved sperm

**Figure 3:**
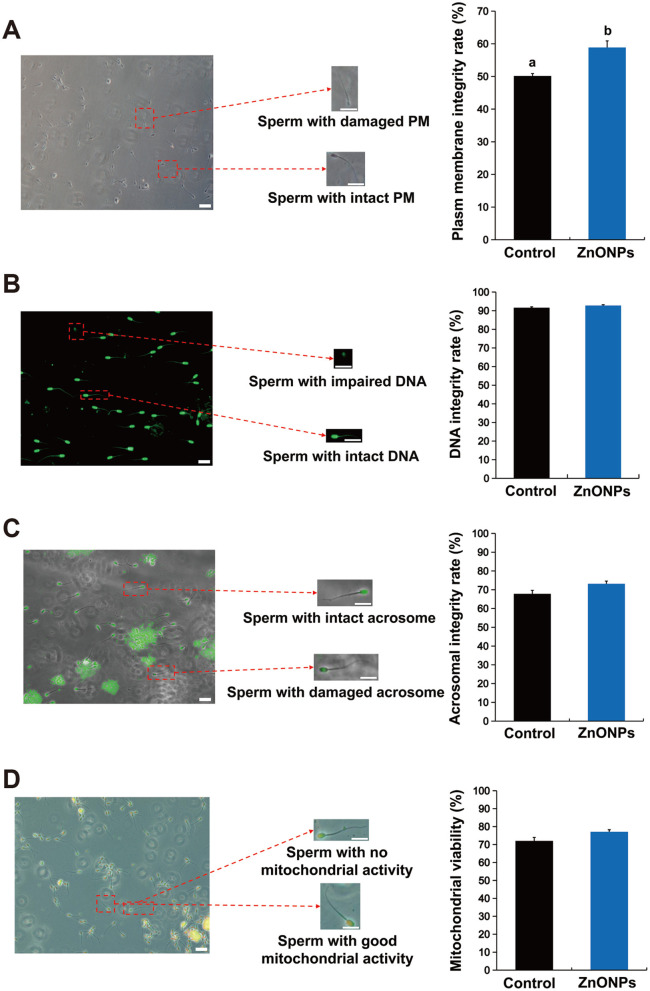
Effects of ZnONPs on the functional characteristics of frozen sperm from Huoshou black boars. **(A)** Plasma membrane integrity. Scale bar: 20 μm. **(B)** DNA integrity. Scale bar: 20 μm. **(C)** Acrosome integrity. Scale bar: 20 μm. **(D)** Mitochondrial activity. Scale bar: 20 μm. The number of biological replicates was 4 boars, with 8 independent ejaculates collected from each. Data were analyzed via Student's *t*-test. Bars with different superscript lowercase letters (a, b) represent significant differences between groups (*P* < 0.05).

Assessment of functional characteristics revealed that plasma membrane integrity was significantly better preserved in sperm treated with 0.1 mg/ml ZnONPs (Figure 3A, *P* < 0.05). However, no significant differences in DNA integrity, acrosome integrity, or mitochondrial activity were detected between the ZnONPs-treated and control groups(Figures 3B-D, *P* > 0.05).

### 3.4 Modulation of oxidative status by ZnONPs in frozen-thawed sperm

**Figure 4:**
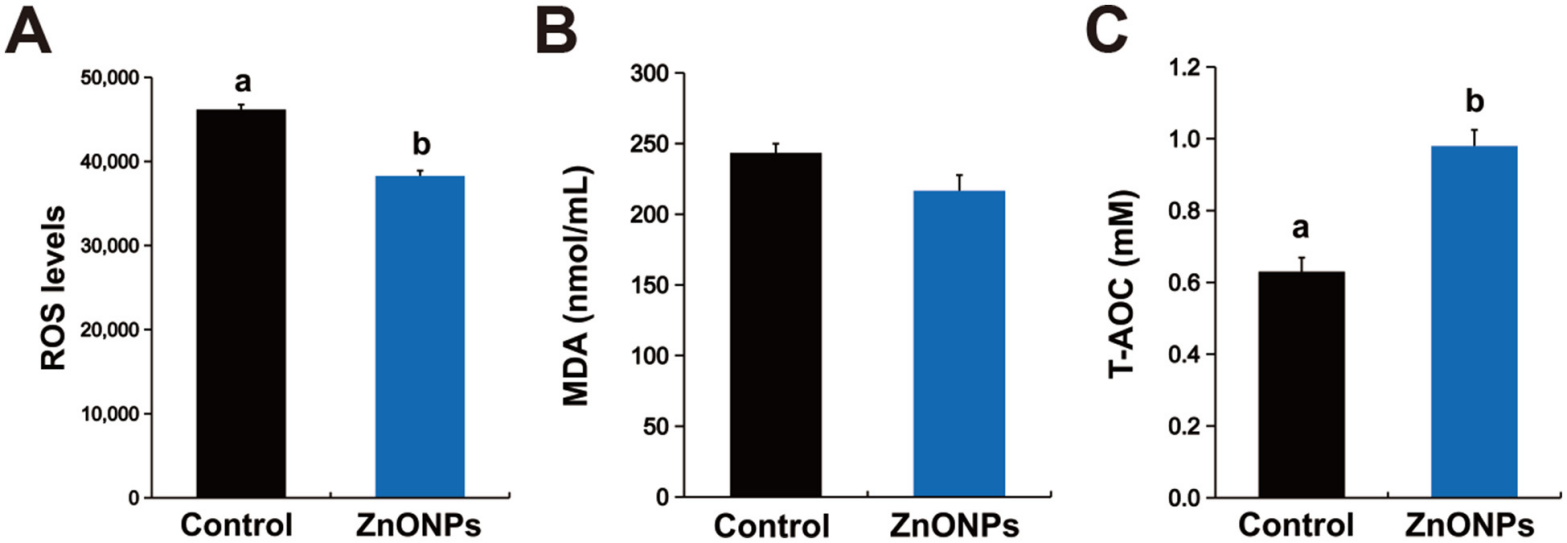
Effects of ZnONPs on oxidative stress-related indicators in frozen sperm. **(A)** ROS levels. **(B)** MDA content. **(C)** T-AOC. The number of biological replicates was 4 boars, with 8 independent ejaculates collected from each. Data were analyzed via Student's *t*-test. Bars with different superscript lowercase letters (a, b) represent significant differences between groups (*P* < 0.05).

ZnONPs exposure notably altered the oxidative stress status of thawed spermatozoa (Figure 4). At 0.1 mg/ml, ZnONPs induced a distinct decrease in ROS levels with a concomitant elevation in T-AOC (Figures 4A, C, *P* < 0.05). MDA, an indicator of lipid peroxidation, showed no significant difference between the treatment and control cohorts (Figure 4B, *P* > 0.05).

### 3.5 Multivariate and metabolite class analysis: ZnONPs vs. the control

**Figure 5:**
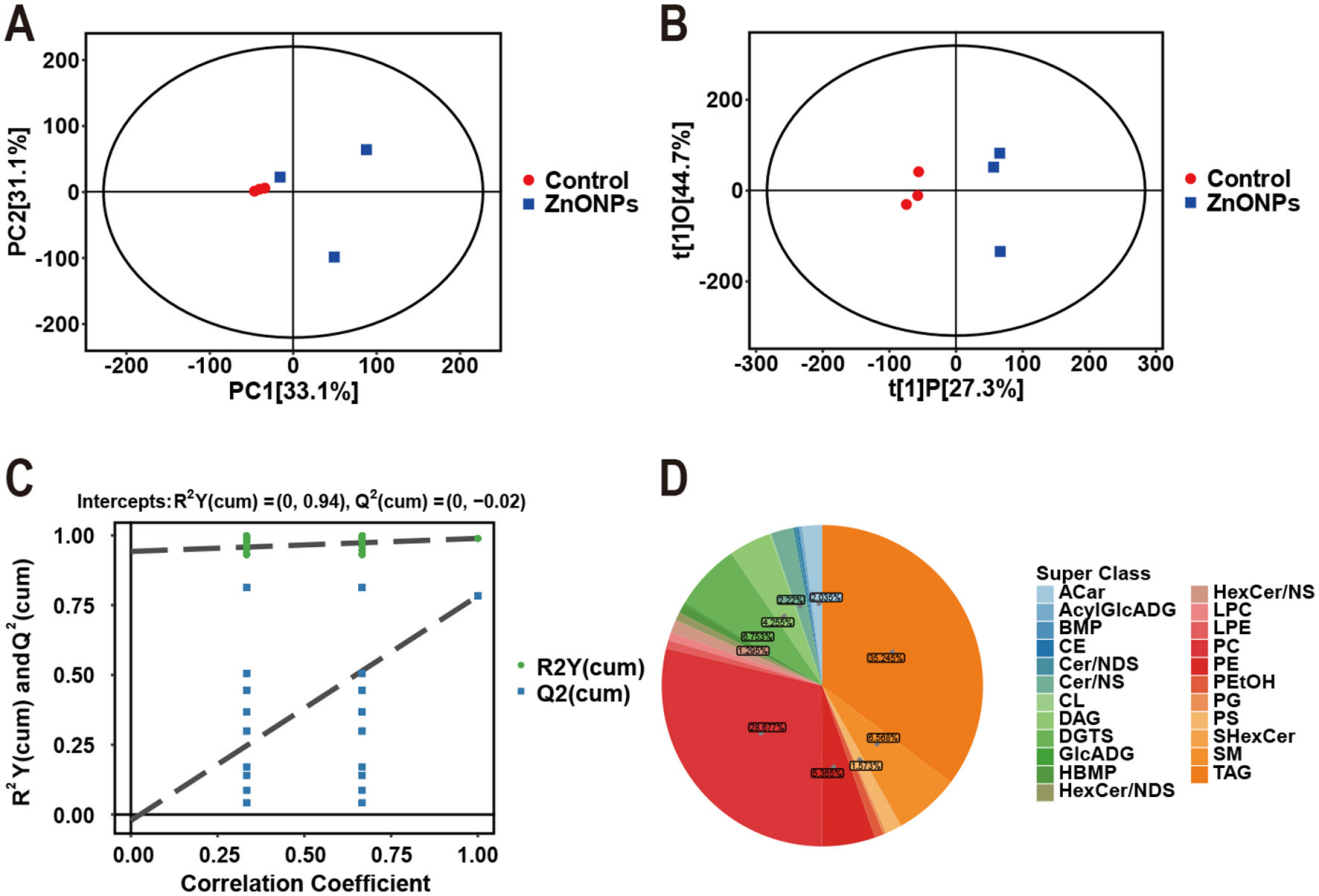
PCA, OPLS-DA and metabolite species analysis between the control and ZnONPs groups. **(A)** PCA score plot in positive ion mode (Control: red; ZnONPs: blue). **(B)** OPLS-DA score plot showing group separation. **(C)** OPLS-DA permutation test plot (R2 > 0, Q2 intercept < 0.05). **(D)** Pie chart showing the classification of identified sperm metabolites in positive ion mode. Data were obtained from *n* = 4 biological replicates (4 independent boars) in each group.

Nontargeted lipidomic profiling was conducted on three sperm samples from each group via positive ion mode. Principal component analysis (PCA) revealed that all samples fell within the 95% confidence interval, confirming the data quality and suitability for subsequent analysis (Figure 5A). Orthogonal projection to latent structure discriminant analysis (OPLS-DA) demonstrated clear segregation of the control from ZnONPs-treated groups, supporting distinct metabolic profiles induced by ZnONPs supplementation (Figure 5B). Permutation testing validated the stability and reliability of the OPLS-DA model. (*R*^2^ near 1, *Q*^2^ < 0) (Figure 5C). The identified metabolites mainly comprised lipids and related lipid-like compounds, including 23 subclasses and 1,081 individual molecules. The most abundant lipid subclasses were triglycerides (TAGs, 35.245%), phosphatidylcholines (PCs, 28.677%), and diacylglyceryltrimethylhomoserines (DGTS, 6.753%) (Figure 5D).

### 3.6 Differential lipid abundance and hierarchical clustering analysis

**Figure 6:**
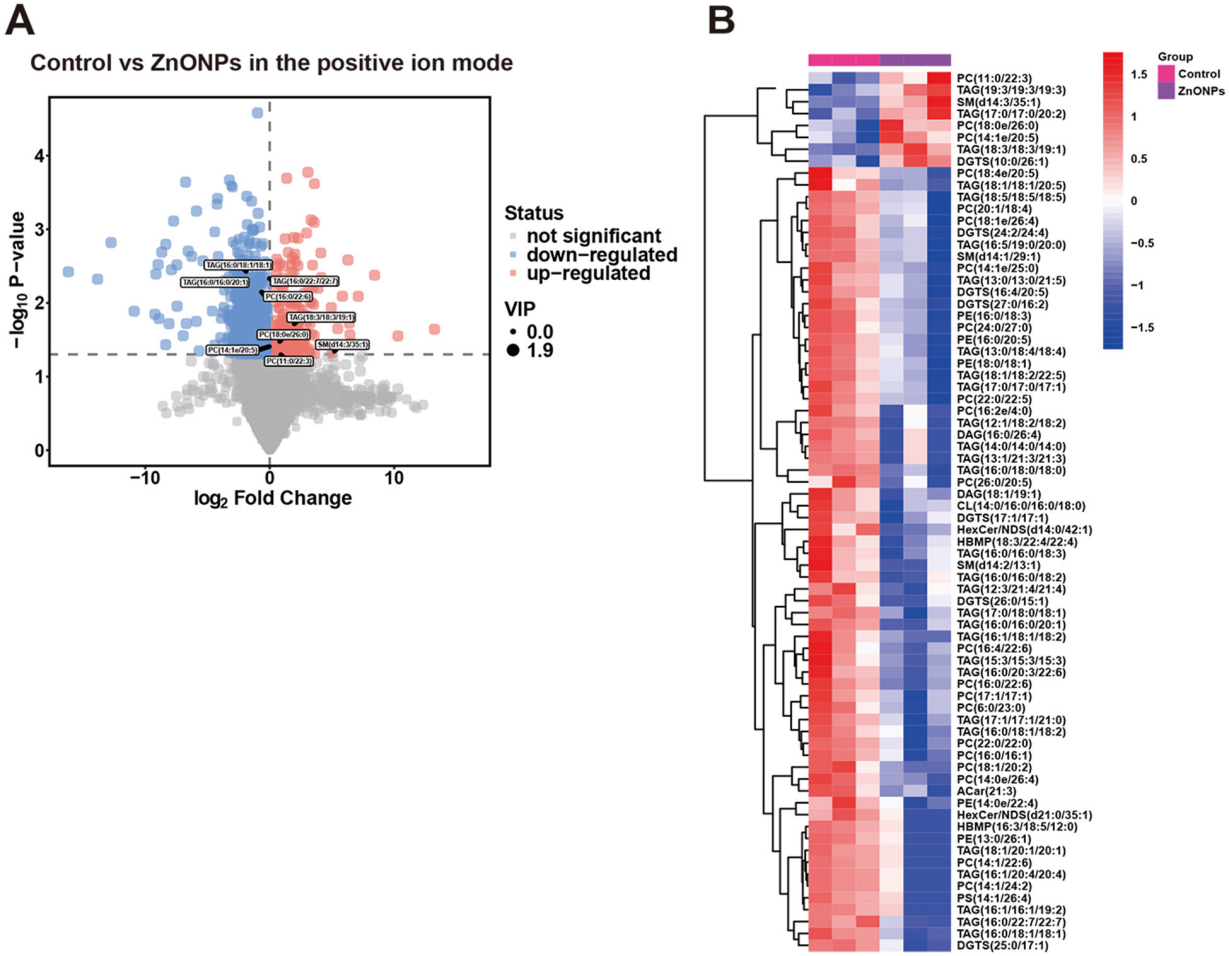
Identification and cluster analysis of differentially abundant metabolites. **(A)** Volcano plot of sperm metabolites in positive ion mode (red: upregulated; blue: downregulated; gray: unchanged). **(B)** Heatmap of sperm metabolites (control: pink; ZnONPs: blue). Data were obtained from *n* = 4 biological replicates (4 independent boars) in each group.

Analysis in positive ion mode identified 1,461 lipids with differential abundance between groups (202 upregulated, 1,259 downregulated) (Figure 6A). Subsequent hierarchical clustering analysis revealed 74 lipid species with statistically significant alterations: 8 were elevated, with 66 being reduced in the ZnONPs group compared with the control group (Figure 6B). The detailed parameters of representative differentially expressed lipids are shown in Table 1. The corresponding heatmap clearly illustrates groupwise clustering and pronounced differences in the abundance patterns of these metabolites.

**Table 1:** Partial differential metabolites screening table (first 2 rows, digits = 2).

MS2_ name	VIP	FC	*P*-VALUE	RT
TAG(17:0/17:0/17:1)	1.1	0.34	0.037	567
SM(d14:2/13:1)	1.5	0.19	0.048	157

VIP, variable importance in the projection; VIP>1, FC, fold change; RT, retention time; TAG, Triacylglycerol; SM, Sphingomyelin; TAG (17:0/17:0/17:1) indicates that this triacylglycerol contains three fatty acid chains. One of the chains consists of 17 carbon atoms and has one double bond, while the other two chains also consist of 17 carbon atoms but do not have double bonds. SM(d14:2/13:1), indicating that sphingosine contains two carbon chains, one of which is composed of 14 carbon atoms forming two double bonds, and the other is composed of 13 carbon atoms forming a double bond.

## 4 Discussion

Cryopreservation enables long-term sperm storage, preserves fertilizing capacity, and facilitates semen transport (30), thereby improving the utilization of superior genetics and enhancing breeding efficiency (31). However, freezing often induces oxidative stress, leading to excessive ROS accumulation, lipid peroxidation, and subsequent quality decline (32–34). Rapid temperature decreases promote ice crystal formation, damaging cells (35), whereas the removal of antioxidant-rich seminal plasma exacerbates oxidative injury (36). Ultralow temperatures also reduce intrinsic antioxidant enzyme activity in spermatozoa (37). As novel antioxidants, ZnONPs possess strong free radical scavenging capacity (38). While their cryoprotective effects have been studied in goats (39), camels (20), and buffaloes (40), data on boar semen are scarce. This study investigated the impact of ZnONPs on the quality and lipid profile of frozen Huoshou black boar semen, aiming to advance porcine semen cryopreservation and support the conservation of this genetic resource.

We first evaluated sperm quality after thawing and incubation at 37 °C with different concentrations. The 0.1 mg/ml concentration had the best cryoprotective effect. Sperm motility, kinematic parameters, and viability were significantly improved with ZnONPs supplementation (*P* < 0.05), which aligns with the findings of Fatemeh Omidi et al. in goats, suggesting that ZnONPs enhance sperm motility during semen preservation.

The plasma membrane integrity and T-AOC significantly increased in the experimental group (*P* < 0.05), whereas the DNA and acrosome integrity remained unchanged. This pattern may be related to the mechanism of action of the ZnONPs. Neutrally charged ZnONPs are unlikely to penetrate the negatively charged plasma membrane of live sperm (41), but may adsorb onto the sperm surface. But may adsorb to the sperm surface (42). We hypothesize that ZnONPs primarily stabilize the sperm plasma membrane and increase the overall antioxidant capacity. As a cofactor for antioxidant enzymes such as superoxide dismutase, zinc can mitigate ROS-induced damage and reduce lipid peroxidation (43). Consistent with these findings, ROS levels were significantly lower in the ZnONPs group. Although the MDA content decreased, the change was not significant. MDA, a terminal product of lipid peroxidation, requires time to accumulate (44). Thus, ZnONPs may protect sperm by interacting with peroxides early in the peroxidation chain or by enhancing SOD activity, thereby impeding lipid peroxidation progression.

Therefore, we used lipidomics to compare frozen sperm from the 0.1 mg/mL ZnONPs group and the control. Analysis revealed 23 lipid subclasses and 1,081 lipid molecules in positive ion mode, predominantly TAG (35.245%), PC (28.677%), and DGTS (6.753%). 74 lipid species were differentially expressed, 8 of which were upregulated and 66 of which were downregulated, confirming that ZnONPs alter the lipid metabolite profile of frozen boar sperm.

The beneficial effects of ZnONPs on frozen semen quality may stem from their high specific surface area and small particle size, allowing efficient interactions with peroxides and mitigating oxidative stress. ZnONPs may also increase intrinsic antioxidant enzyme activity (45), further scavenging excess ROS and reducing lipid peroxidation (46), thereby alleviating cryo-induced oxidative damage. ZnONPs altered the levels of PC, TAG, and DGTS in frozen boar sperm. PC hydrolysis produces lysophosphatidylcholine and polyunsaturated fatty acids, which can promote motility loss and lipid peroxidation (47) TAG provides fatty acids for ATP production and membrane homeostasis (48), whereas DGTS are membrane components that influence fluidity (49). By modulating these metabolites, ZnONPs may improve sperm function and cryopreservation outcomes (50).

Despite these findings, it is important to acknowledge certain limitations of the present study. A key consideration is that the current work was based on *in vitro* experiments, and *in vivo* fertility outcomes were not evaluated. Future studies may therefore focus on *in vivo* trials to further clarify whether ZnONPs can enhance the reproductive performance of frozen-thawed Huoshou black boar semen.

This study demonstrated that ZnONPs can serve as novel exogenous antioxidants in the cryopreservation of Huoshou black boar semen, significantly improving postthaw sperm quality and assisting in the conservation of local genetic resources.

## 5 Conclusion

Supplementing the semen extender with 0.1 mg/ml ZnONPs, a novel antioxidant, effectively increased the postthaw quality of boar semen, and induced significant changes in lipid metabolite expression. These findings provide a theoretical foundation for the development of improved cryoprotectants and support advancements in ultralow-temperature cryopreservation techniques.

## Data Availability

The datasets presented in this study can be found in online repositories. The names of the repository/ repositories and accession number(s) can be found below: https://www.ebi.ac.uk/metabolights/MTBLS12892.
